# Development and Application of a TaqMan-Based One-Step Quadruplex Reverse Transcription Real-Time PCR (RT-qPCR) for Differential Detection of Four Porcine Diarrhea Viruses

**DOI:** 10.1155/tbed/9454210

**Published:** 2025-11-12

**Authors:** Wei Wang, Zhaokun Chen, Dandan Wang, Jizong Li, Baochao Fan, Xuehan Zhang, Min Sun, Yongxiang Zhao, Jinzhu Zhou, Hongqi Shang, Bin Li, Junming Zhou

**Affiliations:** ^1^Institute of Veterinary Medicine, Key Laboratory of Veterinary Biological Engineering and Technology Ministry of Agriculture and Rural Affairs, Jiangsu Academy of Agricultural Sciences, Nanjing 210014, China; ^2^Jiangsu Key Laboratory for Food Quality and Safety, State Key Laboratory Cultivation Base of Ministry of Science and Technology, Nanjing 210014, China; ^3^Jiangsu Co-innovation Center for Prevention and Control of Important Animal Infectious Diseases and Zoonoses, Yangzhou University, Yangzhou 225009, China; ^4^GuoTai (Taizhou) Center of Technology Innovation for Veterinary Biologicals, Taizhou 225300, China; ^5^China Animal Health and Epidemiology Center, Qingdao 266032, China; ^6^Qingdao Lijian Bio-Tech Co., Ltd., Qingdao 266114, China

**Keywords:** porcine deltacoronavirus, porcine epidemic diarrhea virus, porcine rotavirus group A, porcine viral diarrhea, RT-qPCR, transmissible gastroenteritis virus

## Abstract

Porcine epidemic diarrhea virus (PEDV), transmissible gastroenteritis virus (TGEV), porcine deltacoronavirus (PDCoV), and porcine rotavirus group A (PoRVA) are recognized as major enteric viral pathogens responsible for porcine viral diarrhea. These viruses exhibit similar clinical manifestations, including vomiting, diarrhea, and dehydration, which complicate differential diagnosis. Therefore, there is an urgent need for a highly sensitive and specific diagnostic method to differentiate these pathogens. In this study, we developed a TaqMan-based one-step quadruplex reverse transcription real-time PCR (RT-qPCR) assay for the simultaneous and differential detection of these four porcine diarrhea viruses. The standard curves demonstrated correlation coefficients (*R*^2^) exceeded 0.990 across a dynamic range of 10^7.5^ – 10^2.5^ TCID_50_/mL, and amplification efficiency ranged from 90% to 110%. The limit of detection (LOD) were 10^1.5^ TCID_50_/mL. Specificity analysis showed no cross-reactivity with other related pathogens. The assay exhibited good repeatability, with coefficients of variation (CVs) ranging from 0.15% to 1.41% for intra-assay and 0.09% to 2.09% for inter-assay, respectively. Finally, this method was evaluated for its practicality in the field using 348 clinical fecal samples. The positive rates of PEDV, TGEV, PDCoV, and PoRVA were 43.68%, 0.57%, 26.44%, and 33.91%, respectively. Furthermore, the coinfection rates of PEDV/PDCoV, PEDV/PoRVA, PDCoV/PoRVA, and PEDV/PDCoV/PoRVA were14.37%, 13.51%, 2.01%, and 5.75%, respectively. Compared to singleplex RT-qPCR assays, the quadruplex method demonstrated agreement rates ranging from 99.41% to 100% in detecting these four viral pathogens. In conclusion, the developed quadruplex RT-qPCR assay offers a reliable, sensitive, and accurate tool for the identification of four causative agents of porcine viral diarrhea, making it suitable for clinical diagnosis, disease surveillance, and epidemiological studies.

## 1. Introduction

Porcine viral diarrhea is associated with a high mortality rate in piglets, leading to substantial economic losses in the global swine industry [1, 2]. The primary clinical pathogens causing diarrhea in piglets include swine enteric coronaviruses and porcine rotavirus, especially porcine epidemic diarrhea virus (PEDV), transmissible gastroenteritis virus (TGEV), porcine deltacoronavirus (PDCoV), and porcine rotavirus group A (PoRVA) [3, 4]. With the rapid development of intensive animal husbandry, coinfection or secondary infection caused by these pathogens has become increasingly prevalent, resulting in significant morbidity and mortality [2, 5].

PEDV is an enveloped, single-stranded positive-sense RNA virus, which belongs to the *Alphacoronavirus* genus of the *Coronaviridae* family, with a genome approximately 28 kb in length [2]. While pigs of all age groups are susceptible to PEDV infection, the disease causes particularly severe outcomes in piglets, frequently resulting in 100% morbidity and mortality. It was first identified in England in 1971 and isolated in 1978 [6, 7] and has been reported globally [8]. In 2010, a novel mutant strain of PEDV emerged in China, causing nearly 100% morbidity and approximately 80%–100% mortality among neonatal piglets aged under seven days of age [5]. The mutant strain was later detected in the United States in 2013 and subsequently spread to other countries across the Americas, Asia, and Europe, leading to substantial economic losses and becoming a major cause of porcine diarrhea [8, 9]. TGEV is also an enveloped, single-stranded RNA virus belonging to the *Alphacoronavirus* genus in the *Coronaviridae* family, with a genome approximately 28.6 kb in size [10]. It was first reported in the United States in 1946 [11] and has since been identified across the Americas, Europe, Asia, and Africa [12]. TGEV causes rapid weight loss, dehydration, and death in piglets within 1 week of onset, with mortality rates frequently reaching 100%. PDCoV is another enveloped, single-stranded RNA virus that belongs to the *Deltacoronavirus* genus in the family *Coronaviridae*, with a genome approximately 25.4 kb in length [13]. It was initially identified in Hong Kong in 2012 [14] and has since rapidly spread to multiple countries, causing significant economic losses in the swine industry, with mortality rates ranging from 30% to 40% [15]. PoRV is a nonenveloped, double-stranded RNA virus classified under the genus *Rotavirus* in the *Reoviridae* family [4], with a genome of approximately 18.5 kb composed of 11 dsRNA segments that encode six structural proteins (VP1-VP4, VP6, and VP7) [16]. Based on antigenic differences in the VP6 protein, PoRV is classified into 10 serogroups (A–J), among which groups A, B, C, E, and H have been detected in swines [16]. Among these, PoRVA has emerged as the primary rotavirus causing gastrointestinal disease in pigs, with high prevalence and pathogenicity since its initial isolation from infected pigs in 1976 [16, 17].

Piglets infected with PEDV, TGEV, PDCoV, or PoRVA typically exhibit similar clinical symptoms, such as vomiting, diarrhea, and dehydration [18, 19]. Moreover, coinfections and secondary infections involving these viruses are highly prevalent, making clinical differentiation challenging [1, 2]. Therefore, there is an urgent need for a highly sensitive and specific method to differentiate these viral pathogens. Real-time PCR (qPCR), which monitors the amplification process through the real-time detection of fluorescence signals, is a rapid, accurate, and sensitive technique for pathogen detection and quantification [4]. Compared to conventional singleplex RT-qPCR, multiplex RT-qPCR can simultaneously detect multiple pathogens within a single reaction system using extracted viral RNA, offering advantages in terms of cost-effectiveness, high efficiency, and high-throughput capability. In this study, primers and probes for RT-qPCR were designed based on conserved fragments of the *N* gene of PEDV, TGEV, and PDCoV, as well as the VP6 gene of PoRVA. Subsequently, a TaqMan-based one-step quadruplex RT-qPCR assay was successfully developed, and its performance was evaluated using clinical samples.

## 2. Materials and Methods

### 2.1. Viruses and Viral Nucleic Acids

PEDV/AH2012/12 strain (GenBank: KU646831.1) was cultured in Vero cells with a titer of 10^7.0^ TCID_50_/mL; TGEV/JS2012 strain (GenBank: KT696544) was cultured in ST cells with a titer of 10^7.0^ TCID_50_/mL; PDCoV/CZ2020 strain (GenBank: OK546242) was cultured in LLC-PK1 cells with a titer of 10^7.0^ TCID_50_/mL; and PoRVA/JSJR2023 strain (GenBank: PP100149.1-PP100159.1) was cultured in MA104 cells with a titer of 10^7.0^ TCID_50_/mL. These four viruses were isolated and conserved in our laboratory. In addition, the nucleic acids (DNA or RNA) of porcine reproductive and respiratory syndrome virus (PRRSV), classical swine fever virus (CSFV), pseudorabies virus (PRV), porcine parvovirus (PPV), porcine circovirus type 2 (PCV2), getah virus (GETV), porcine sapelovirus (PSV), porcine teschovirus (PTV), and porcine norovirus (PoNoV) were preserved in our laboratory. All viral samples and nucleic acid extracts were stored at –80 °C.

### 2.2. Primers and TaqMan Probes Design

At least 20 genome sequences each of PEDV, TGEV, PDCoV, and PoRVA were downloaded from the NCBI database for analysis. The most conserved regions of the *N* gene in PEDV, TGEV and PDCoV, as well as the VP6 gene in PoRVA, were identified using DNASTAR software (version 7.0). Primers and probes were subsequently designed using Primer Premier 5 software (Premier, Canada) based on these conserved regions. In certain sites of the VP6 gene in PoRVA, base mutations were observed. To enhance the detection efficiency, degenerate bases (R, H, and Y) were incorporated into the design. TaqMan probes for PEDV, TGEV, PDCoV, and PoRVA were labeled with FAM, ROX, Cy5, and VIC at the 5′-end, respectively, with BHQ1-BHQ3 used as 3′-end quenchers. The sequences of the primers and probes designed in this study are listed in Table 1 and were synthesized by Sangon Biotech (Shanghai) Co., Ltd.

### 2.3. Preparation of Viral RNA Standard Mixtures

A known value of 20 mL of each of the four viruses was used for total RNA extraction with the Viral DNA/RNA Extraction Maxi Kit (ONREW, China) and 6 mL RNA was eluted and stored in RNA storage solution (ThermoFisher Scientific, USA) at –80 °C until used. The final RNA concentration of the four viruses corresponded to a virus titer of 10^7.5^ TCID_50_/mL. The four viral RNA samples were then mixed at a 1:1:1:1 ratio, with one portion of RNA storage solution, indicating that all viruses were diluted 5-fold, which designated as viral RNA standard mixtures (10^7.5^ TCID_50_/mL). Then the standard mixtures (10^7.5^ TCID_50_/mL) were serially diluted 10-fold using EASY Dilution (Takara, China) to achieve final RNA concentrations corresponding to virus titers ranging from 10^6.5^ to 10^0.5^ TCID_50_/mL.

### 2.4. Establishment of Standard Curves for One-Step Quadruplex RT-qPCR

All one-step quadruplex RT-qPCR assays were performed using an ABI7500 real-time PCR system (ThermoFisher Scientific, USA) to determine the optimal reaction conditions (see Table S1). Following repeated experimental trials, the optimized reaction conditions for the quadruplex RT-qPCR assay were determined as follows: 5.0 µL of 5 × One Step U^+^ Mix Buffer (Vazyme, China), 1.25 µL of One Step U^+^ Enzyme Mix (Vazyme, China), 0.4 µL each of forward and reverse primers (20 µM), 0.2 µL each of probe (20 µM), 5.0 µL of RNA template, and 9.75 µL of nuclease-free water, yielding a total reaction volume of 25.0 µL. The amplification was carried out using the following thermal cycling program: 55 °C for 15 min; 95 °C for 30 s; followed by 40 cycles of 95°C for 10 s and 60°C for 34 s. Fluorescence signals were automatically collected at the end of each amplification cycle. Based on the optimized reaction conditions and thermal cycling protocol, two replicates of viral RNA standard mixtures (ranging from 10^7.5^ to 10^2.5^ TCID_50_/mL) were tested using the quadruplex RT-qPCR assay to generate standard curves, which were derived from linear regression analysis between cycle threshold (*C*_t_) values and the logarithm of viral RNA concentration.

### 2.5. Specificity Analysis

To evaluate the specificity of the developed RT-qPCR assay, nucleic acid samples of PRRSV, CSFV, PRV, PPV, PCV2, GETV, PSV, PTV, and PoNoV were used as templates. Viral RNA standard mixtures at a concentration of 10^6.5^ TCID_50_/mL were used as positive controls, and nuclease-free water was included as a negative control.

### 2.6. Sensitivity Analysis

Ten-fold serial dilutions of the viral RNA standard mixtures, ranging from 10^7.5^ to 10^0.5^ TCID_50_/mL, were used as templates for amplification to determine the limit of detection (LOD) of the developed RT-qPCR assay.

### 2.7. Repeatability Analysis

The repeatability of the RT-qPCR assay was evaluated by calculating the coefficients of variation (CVs) for both intra-assay and inter-assay. Viral RNA standard mixtures of 10^6.5^, 10^4.5^,10^2.5^ TCID_50_/mL were used as templates. For intra-assay repeatability, each template was tested in triplicate within a single experimental run. For inter-assay repeatability, each template was analyzed in three independent assays performed on separate days.

### 2.8. Clinical Sample Detection

A total of 348 fecal samples were collected from multiple provinces across China, including Jiangsu (184 samples), Shanghai (41 samples), Anhui (23 samples), Henan (47 samples), Sichuan (18 samples), and Hunan (35 samples), between May 2023 and June 2025. All collected samples were obtained from pig farms experiencing piglet diarrhea or vomiting outbreaks. Clinical samples were resuspended in phosphate-buffered saline (PBS), and the supernatant was obtained following vortexing and centrifugation. Viral nucleic acids were extracted using the Magbead Viral DNA/RNA Kit (Auto Plate) (CWBIO, China). The viral RNA standard mixtures at a concentration of 10^6.5^ TCID_50_/mL served as positive control, while nuclease-free water was used as negative control. Under optimized reaction conditions, the quadruplex RT-qPCR assay was performed to detect the presence of each target pathogen. The infection rates were calculated based on the assay results of all clinical samples. Concurrently, these samples were also tested by the singleplex RT-qPCR assay for PEDV [20], TGEV [21], PDCoV [22], and PoRVA [17], enabling a comparative evaluation of the concordance rate between the two detection methods.

## 3. Results

### 3.1. Establishment of the One-Step Quadruplex RT-qPCR

**Figure 1:**
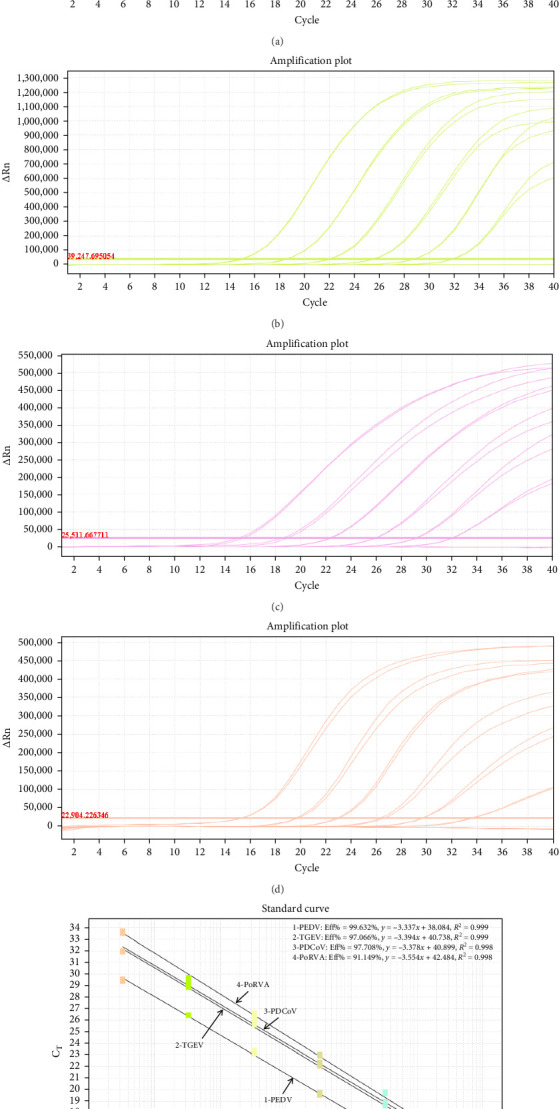
Amplification curves and standard curve of the one-step quadruplex RT-qPCR assay. The amplification curves were generated using viral RNA standard mixtures of PEDV (A), TGEV (B), PDCoV (C), and PoRVA (D) at concentrations ranging from 10^7.5^ to 10^2.5^ TCID_50_/mL. The standard curves (E) showed an excellent linear relationship (*R*^2^ ≥ 0.990) between RNA template concentration and the corresponding *C*_t_ values.

Ten-fold serial dilutions of viral RNA standard mixtures were analyzed using the optimized multiplex assay. The results demonstrated that the one-step quadruplex RT-qPCR assay successfully detected all target genes of the four viruses based on the amplification curves (Figure 1A–D). The standard curves exhibited excellent correlation coefficients (*R*^2^) and amplification efficiency (Eff%) for each virus (Figure 1E), with PEDV (*R*^2^ = 0.999; Eff% = 99.362%), TGEV (*R*^2^ = 0.999; Eff% = 97.066%), PDCoV (*R*^2^ = 0.998; Eff% = 97.708%), and PoRVA (*R*^2^ = 0.998; Eff% = 91.149%), respectively. These results indicated establishment of the quadruplex RT-qPCR assay was valid and reliable [23].

### 3.2. Specificity Analysis

**Figure 2:**
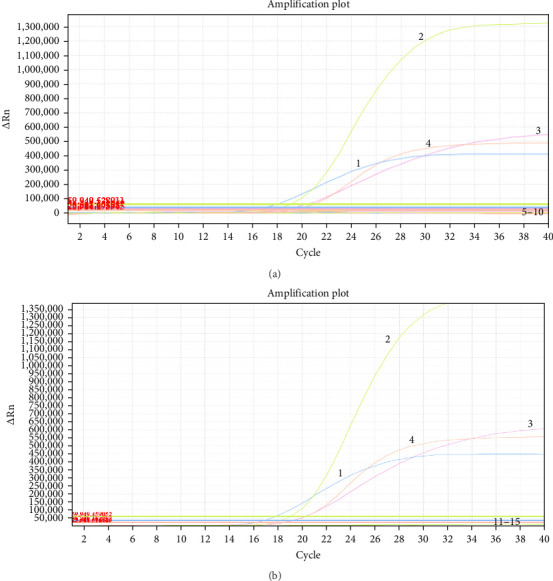
Specificity analysis of the one-step quadruplex RT-qPCR assay. (A) 1: PEDV; 2: TGEV; 3: PDCoV; 4: PoRVA; 5–9: PRRSV, CSFV, PRV, PPV, PCV2; 10: Negative control. (B) 1: PEDV; 2: TGEV; 3: PDCoV; 4: PoRVA; 11–14: GETV, PSV, PTV, PoNoV; 15: Negative control. Specific amplification curves were observed exclusively for PEDV, TGEV, PDCoV, and PoRVA.

To evaluate the specificity of the RT-qPCR assay, nucleic acid-positive samples of PRRSV, CSFV, PRV, PPV, PCV2, GETV, PSV, PTV, or PoNoV were used as templates for amplification with this multiplex system. The results demonstrated that only PEDV, TGEV, PDCoV, and PoRVA produced amplified signals and generated specific amplification curves, whereas no positive signals or amplification curves were observed for PRRSV, CSFV, PRV, PPV, PCV2, GETV, PSV, PTV, or PoNoV (Figure 2A, B), indicating the high specificity of the developed RT-qPCR assay.

### 3.3. Sensitivity Analysis

**Figure 3:**
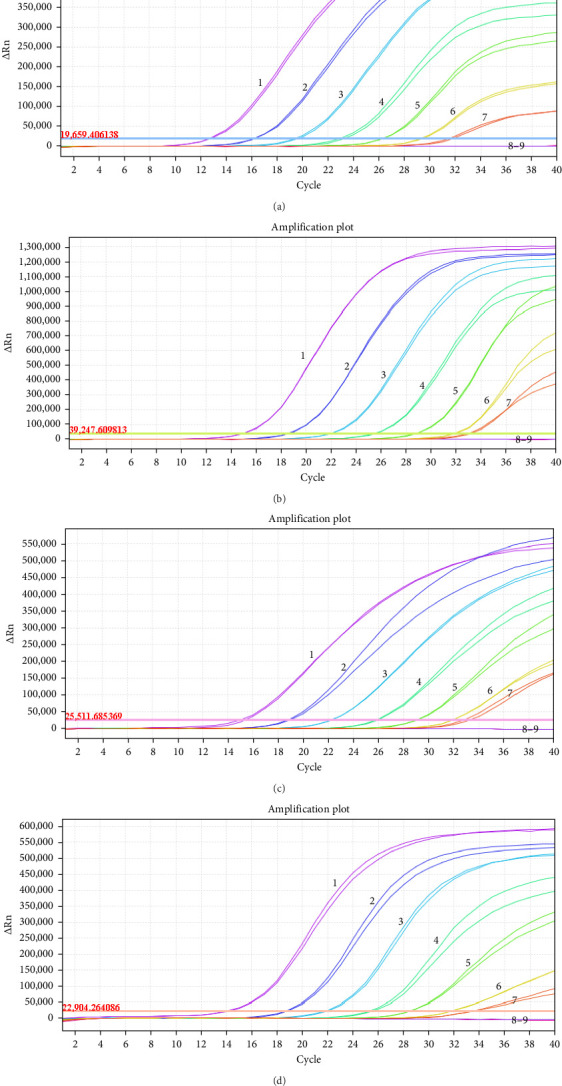
Sensitivity analysis. 1–8: 10^7.5^ to 10^0.5^ TCID_50_/mL of viral RNA standard mixtures of PEDV (A), TGEV (B), PDCoV (C), and PoRVA (D); 9: Negative control.

Ten-fold serial dilutions of viral RNA standard mixtures ranging from 10^7.5^ to 10^0.5^ TCID_50_/mL were used to determine the LOD of the developed RT-qPCR. The LOD for each virus was 10^1.5^ TCID_50_/mL (Figure 3), indicating the high sensitivity of the developed RT-qPCR assay.

### 3.4. Repeatability Analysis

To estimate the repeatability of the developed RT-qPCR assay, three viral RNA standard mixtures were used to assess the intra-assay and inter-assay variation. The results showed that the CVs of *C*_t_ values of intra-assay and inter-assay ranged from 0.15% to 1.41% and from 0.09% to 2.09%, respectively (Table 2), indicating the excellent repeatability of the asaay.

### 3.5. Clinical Application of the Quadruplex RT-qPCR Assay

**Figure 4:**
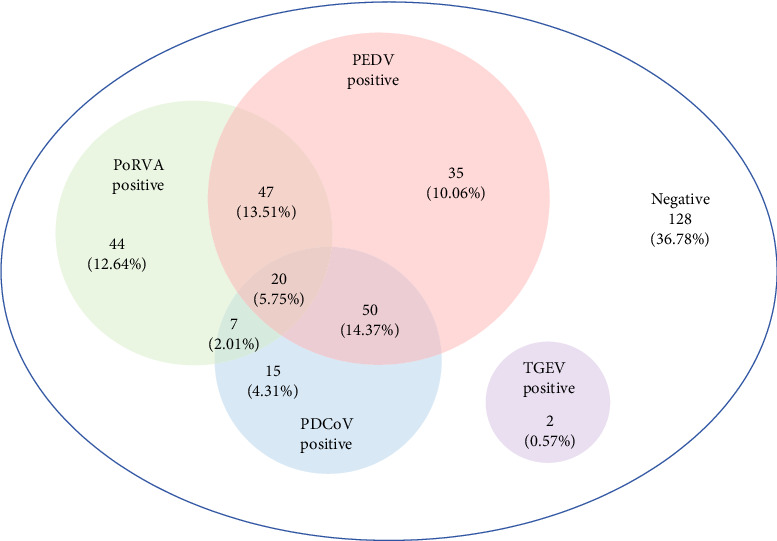
Coinfection of the porcine diarrhea viruses by the quadruplex RT-qPCR assay.

The developed RT-qPCR assay was applied to analyze the 348 clinical fecal samples collected from numerous provinces across China. The positive rates of PEDV, TGEV, PDCoV, and PoRVA were 43.68% (152/348), 0.57% (2/348), 26.44% (92/348), and 33.91% (118/348), respectively (Table 3). Furthermore, the coinfection rates for PEDV/PDCoV, PEDV/PoRVA, PDCoV/PoRVA, and PEDV/PDCoV/PoRVA were 14.37% (50/348), 13.51% (47/348), 2.01% (7/348), and 5.75% (20/348), respectively (Figure 4). However, no coinfection involving TGEV and any of the other three viruses were observed.

All 348 clinical samples were also analyzed using the singleplex RT-qPCR assays, and the positive rates of PEDV, TGEV, PDCoV, and PoRVA were 43.10% (150/348), 0.57% (2/348), 26.15% (91/348), and 33.91% (118/348), respectively. The results demonstrated that the coincidence rates between the two methods were 99.43%, 100%, 99.71%, and 100%, respectively (Table 3).

## 4. Discussion

Porcine viral diarrhea is a prevalent clinical disease that causes substantial economic losses in the swine industry. Especially, PEDV, TGEV, PDCoV, and PoRVA are recognized as major porcine enteric viral pathogens responsible for this disease [1, 24]. With the expansion of large-scale intensive swine farming, coinfections involving these four pathogens have become increasingly common on pig farms [2]. Rapid, accurate detection and identification of these pathogens play a critical role in preventing and controlling the spread of infectious diseases. Given the similarities in clinical symptoms and pathological manifestations, coupled with the high prevalence of coinfection among these four pathogens, it is extremely difficult to distinguish them solely based on clinical diagnosis [2, 25]. Therefore, there is an urgent need for a rapid and reliable diagnostic method to simultaneously differentiate these four pathogens.

In this study, specific primers and probes for the RT-qPCR assay were designed for the conserved regions of the *N* gene of PEDV, TGEV, PDCoV, and the VP6 gene of PoRVA. The correlation coefficients (*R*^2^) of standard curves exceeded 0.990 in the range 10^7.5^ to 10^2.5^ TCID_50_/mL, and amplification efficiency ranged from 90% to 110%, indicating the amplification reaction was well optimized. Following optimizing the amplification conditions, the one-step quadruplex RT-qPCR method was successfully developed to simultaneously detect the porcine diarrhea viruses in a single reaction system using extracted viral RNA. The method shows high specificity without cross-reaction with other swine pathogens (PRRSV, CSFV, PRV, PPV, and PCV2) and demonstrated high sensitivity with LOD of 10^1.5^ TCID_50_/mL. Additionally, the RT-qPCR assay showed excellent repeatability, with CVs ranging from 0.15% to 1.41% for intra-assay and 0.09% to 2.09% for inter-assay.

A total of 348 clinical samples exhibiting diarrhea symptoms were used to compare the results of the singleplex RT-qPCR assay [17, 20, 21, 26] and our newly developed multiplex RT-qPCR method for detecting four porcine diarrhea viruses. The two methods showed almost agreement (coincidence rate > 99%), indicating that the singleplex RT-qPCR assay could be replaced by the multiplex RT-qPCR assay developed in this study for the simultaneous differentiation of these four porcine diarrhea viruses. Among these 348 samples, 128 were negative and 220 were positive for single or coinfections of these four viruses. The positive rates of PEDV, TGEV, PDCoV, and PoRVA were 43.68%, 0.57%, 26.44%, and 33.91%, respectively, suggesting that PEDV remains the primary pathogen of porcine diarrhea, while the relatively high infection rates of PoRV and PDCoV should also be highly concerned. Numerous studies have reported that PEDV, PoRV, PDCoV, and TGEV were common in many pig herds in China [2, 5, 24, 27]. Furthermore, the positive rate of TGEV was very low at 0.57%, consistent with previously reported prevalence rate ranging from 0.2% [18] to 3.91% [4]. This suggests that TGEV may have been partially replaced by porcine respiratory coronavirus (PRCV), a TGEV mutant with a spike (*S*) gene deletion that alters tissue tropism [28]. Notably, infection rate of PRCV has reached 11.8%, meaning that PRCV infection is prevalent in swine farms across China [29].

Previous studies have reported that coinfection with these four viruses is also a common occurrence in clinical settings [2, 5, 30, 31]. In our study, the results showed that the coinfections of PEDV/PDCoV and PEDV/PoRVA were the most prevalent, with positive rates reaching 14.37% and 13.51%, respectively, which is in agreement with previous findings [27, 30, 31]. The positive rate of triple infection with PEDV/PDCoV/PoRVA was as high as 5.75%, indicating that the pathogens causing viral diarrhea in pig farms are complex and diverse.

## 5. Conclusions

PEDV, TGEV, PDCoV, and PoRVA are important porcine diarrhea viruses that seriously threaten the swine industry worldwide. In this study, a one-step quadruplex RT-qPCR assay was developed for the simultaneous and differential detection of these four viral pathogens, with excellent specificity, high sensitivity, strong repeatability, and convenient operation. This method provides a reliable tool for accurate diagnosis and epidemiological investigations in laboratory settings.

## Figures and Tables

**Table 1:** Primers and probes designed for the quadruplex RT-qPCR.

Virus	Primer/ probe	Sequence (5′→3′)	Size (bp)	Target gene
PEDV	Forward	GGCATTTCTACTACCTCGGA	90	N
	Reverse	CGCCTTCTTTAGCAACCCAG		
	Probe	FAM-ACCTCACGCCGACCTCCGCT-BHQ1		

TGEV	Forward	GCAGTTCAGCCAATTTTGGTGAC	88	N
	Reverse	GATGGAACACATTCAGCCAGTTG		
	Probe	ROX-TGGCACTGCTCCCATTGGCAACG-BHQ2		

PDCoV	Forward	CAGACATGTGCCTGGTGTT	71	N
	Reverse	TTGCCCCTGCCTGAAAGTT		
	Probe	Cy5-TGCTTTTCGCTGGCCACCTTGA-BHQ3		

PoRVA	Forward	GACTTACRTTRCGHATTGAATCTG	79	VP6
	Reverse	GTYACATTTGCCAAYAAAGTTTC		
	Probe	VIC-CAGTTTGTGAATCTGTGCTTGCGGA-BHQ1		

**Table 2:** The repeatability analysis of the one-step quadruplex RT-qPCR.

Virus	Titer(TCID_50_/mL)	Intra-assay			Inter-assay		
		Ct Mean	SD	CV (%)	Ct Mean	SD	CV (%)
PEDV	10^6.5^	16.22	0.17	1.02	15.89	0.26	1.66
	10^4.5^	23.08	0.25	1.08	22.64	0.47	2.09
	10^2.5^	29.41	0.10	0.34	29.22	0.10	0.36

TGEV	10^6.5^	18.62	0.07	0.37	18.72	0.35	1.88
	10^4.5^	25.56	0.06	0.22	25.51	0.35	1.36
	10^2.5^	31.84	0.17	0.55	32.02	0.15	0.47

PDCoV	10^6.5^	18.83	0.20	1.06	18.57	0.29	1.58
	10^4.5^	25.86	0.14	0.55	25.36	0.49	1.91
	10^2.5^	31.93	0.21	0.65	31.74	0.27	0.86

PoRVA	10^6.5^	18.55	0.10	0.55	18.77	0.02	0.09
	10^4.5^	25.51	0.04	0.15	25.40	0.12	0.49
	10^2.5^	31.59	0.44	1.41	31.84	0.60	1.87

**Table 3:** Detection of clinical samples by the quadruplex RT-qPCR and singleplex RT-qPCR assays.

Virus	Quadruplex RT-qPCR (in this study)			Singleplex RT-qPCR			Coincidence rate (%)
	Sample number	Positive number	Positive rate (%)	Sample number	Positive number	Positive rate (%)	
PEDV	348	152	43.68	348	150	43.10	99.43
TGEV	348	2	0.57	348	2	0.57	100
PDCoV	348	92	26.44	348	91	26.15	99.71
PoRVA	348	118	33.91	348	118	33.91	100

## Data Availability

The data that support the findings of this study are available from the corresponding author upon reasonable request.
