# Supplementary material for: Development and Application of a TaqMan-Based One-Step Quadruplex Reverse Transcription Real-Time PCR (RT-qPCR) for Differential Detection of Four Porcine Diarrhea Viruses
Source: Transbound Emerg Dis. 2025 Nov 12;2025:9454210. doi: 10.1155/tbed/9454210 (PMC12643720; doi:10.1155/tbed/9454210)
Supplement: Supporting Information — Additional supporting information can be found online in the Supporting Information section. [file 9454210.f1.docx]

Table S1 Optimization of forward/reverse primer and probe concentrations

| The volume (µL) of forward / reverse primers and probes | PEDV | | TGEV | | PDCoV | | PoRV | |
| --- | --- | --- | --- | --- | --- | --- | --- | --- |
|  | Ct Mean | SD | Ct Mean | SD | Ct Mean | SD | Ct Mean | SD |
| Group 0.1/0.1/0.05 | 18.60 | 0.11 | 23.76 | 0.08 | 21.93 | 0.08 | 22.03 | 0.17 |
| Group 0.2/0.2/0.1 | 18.16 | 0.17 | 22.61 | 0.04 | 21.40 | 0.02 | 21.07 | 0.09 |
| Group 0.4/0.4/0.2 | 17.59 | 0.09 | 21.76 | 0.05 | 20.92 | 0.04 | 20.52 | 0.03 |
| Group 0.8/0.8/0.4 | 17.42 | 0.06 | 21.32 | 0.07 | 20.71 | 0.03 | 20.49 | 0.09 |
